# Evaluation of the Interactions between Mumps Virus and Guinea Pig

**DOI:** 10.1128/jvi.00359-23

**Published:** 2023-04-05

**Authors:** Maja Lang Balija, Adela Štimac, Tanja Košutić Gulija, Andrea Gudan Kurilj, Ana Bekavac, Ante Plećaš, Beata Halassy, Maja Jagušić, Dubravko Forčić

**Affiliations:** a Centre for Research and Knowledge Transfer in Biotechnology, University of Zagreb, Zagreb, Croatia; b Center of Excellence for Viral Immunology and Vaccines, CERVirVac, Zagreb, Croatia; c Department of Veterinary Pathology, Faculty of Veterinary Medicine, University of Zagreb, Zagreb, Croatia; d Laboratory for Stem Cells, School of Medicine, Zagreb, Croatia; e Department of Anatomy, Histology and Embryology, Faculty of Veterinary Medicine, University of Zagreb, Zagreb, Croatia; St. Jude Children’s Research Hospital

**Keywords:** guinea pig, host-pathogen interaction, mumps virus, primary cell culture, sialic acid

## Abstract

Mumps is a highly contagious viral disease that can be prevented by vaccination. In the last decade, we have encountered repeated outbreaks of mumps in highly vaccinated populations, which call into question the effectiveness of available vaccines. Animal models are crucial for understanding virus-host interactions, and viruses such as mumps virus (MuV), whose only natural host is the human, pose a particular challenge. In our study, we examined the interaction between MuV and the guinea pig. Our results present the first evidence that guinea pigs of the Hartley strain can be infected *in vivo* after intranasal and intratesticular inoculation. We observed a significant viral replication in infected tissues up to 5 days following infection and induction of cellular and humoral immune responses as well as histopathological changes in infected lungs and testicles, without clinical signs of disease. Transmission of the infection through direct contact between animals was not possible. Our results demonstrate that guinea pigs and guinea pig primary cell cultures represent a promising model for immunological and pathogenetic studies of the complex MuV infection.

**IMPORTANCE** Understanding of mumps virus (MuV) pathogenesis and the immune responses against MuV infection is limited. One of the reasons is the lack of relevant animal models. This study explores the interaction between MuV and the guinea pig. We demonstrated that all tested guinea pig tissue homogenates and primary cell cultures are highly susceptible to MuV infection and that α2,3-sialylated glycans (MuV cellular receptors) are being abundantly expressed at their surface. The virus remains in the guinea pig lungs and trachea for up to 4 days following intranasal infection. Although asymptomatic, MuV infection strongly activates both humoral and cellular immune response in infected animals and provides protection against virus challenge. Infection of the lungs and testicles after intranasal and intratesticular inoculation, respectively, is also supported by histopathological changes in these organs. Our findings give perspective for application of guinea pigs in research on MuV pathogenesis, antiviral response, and vaccine development and testing.

## INTRODUCTION

Mumps is an acute, highly infectious viral disease. *Mumps orthorubulavirus* (MuV) belongs to the genus *Orthorubulavirus* of the family *Paramyxoviridae*, which includes enveloped, nonsegmented, negative-strand RNA viruses (1). Two MuV envelope glycoproteins, hemagglutinin-neuraminidase (HN) and fusion (F) protein, are involved in receptor binding and mediate membrane fusion with target cells (2, 3). HN specifically recognizes sialic acid (SA)-containing glycoconjugate structures present on the host cells, preferring unbranched α2,3-sialylated glycans, which therefore play a key role in virus entry into host cells and infectivity (4).

The primary site of virus replication is the epithelium of the upper airway mucosa. After crossing the epithelial barrier, the virus accesses the regional lymph nodes and exploits mononuclear cells for further dissemination (5).

Lymphocytic infiltration and destruction of periductal cells lead to obstruction of the ducts in the salivary glands and seminal tubules of the testes, thus inducing the classic symptoms of MuV infection (6). Virus is excreted in the saliva from approximately 1 week before to 1 week after the onset of salivary gland swelling, although MuV has also been identified in the saliva of asymptomatic persons (6). In addition, the virus can be excreted in the urine at least for 10 to 14 days (7).

Among serious complications of mumps in childhood is meningoencephalitis (6, 8), which can be fatal (6), as well as orchitis, which occurs in all age groups and can lead to fertility disorders and even sterility. In addition, the inflammation of other organs (pancreas, thyroid, ovaries, kidneys, and heart muscle) is frequently associated with MuV infection (9).

Following the introduction of the mumps vaccine in the routine vaccination of children (two-dose measles-mumps-rubella [MMR] vaccine), the occurrence of mumps has been drastically reduced. However, in recent years, an increased number of mumps outbreaks has been observed in vaccinated populations (10), one likely reason being an ineffective immune response to available vaccines (11–13). Also, epidemiological studies have shown that certain mumps vaccines are not completely harmless and can lead to significant neurological side effects (14).

The reemergence of MuV infections makes it worthwhile to review the complex virology of mumps and immunity achieved after vaccination, as well as the prospects for developing MuV vaccines with enhanced immunity in the future (11). Unfortunately, understanding of MuV pathogenesis and the immune responses required for protection against MuV infection is limited, one of the reasons being the lack of adequate and relevant animal models (15).

The search for an animal model for mumps has been going on for decades. Experimentally, only primates can show symptoms of the disease after MuV infection (16). Monkeys are evolutionarily closer to humans, but they are extremely expensive to breed, and it is impossible to secure them in sufficient numbers for basic research. Therefore, researchers resort to more affordable animal species such as cats, dogs, guinea pigs, cotton rats, hamsters, ferrets, rabbits, and rats, as well as various mouse models (16–19). Each animal model comes with its own unique set of advantages and limitations; nevertheless, the biggest problem arises when investigating the viruses to which animals are not susceptible.

In our study, we aimed to investigate the interaction between guinea pigs and MuV from different aspects. First, we investigated the possibility of replication of wild-type, vaccine, and recombinant MuV strains within different tissue homogenates and primary cell cultures of guinea pigs (*in vitro* condition). Then, we evaluated the susceptibility of guinea pigs to intranasal (i.n.) and intratesticular (i.t.) MuV infection (*in vivo* condition). We monitored the sites of virus persistence and virus excretion in tissues and in body secretions and/or fluids following different routes of infection, the development of cellular or humoral immune responses, and virus pathology in infected tissues as well as the possibility of virus transmission among animals.

## RESULTS

### Infection of guinea pig tissue homogenates by various MuV strains.

**FIG 1:**
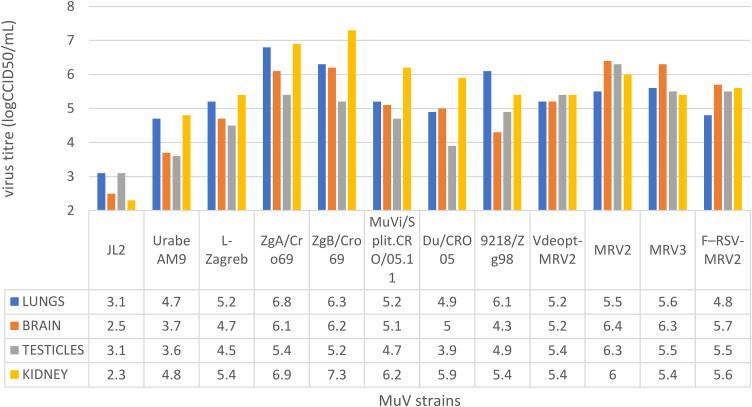
Virus titer after infection of guinea pig tissue homogenates by various MuV strains: JL2, Urabe AM9, L-Zagreb, ZgA/Cro69, ZgB/Cro69, MuVi/Split.CRO/05.11, Du/CRO05, 9218/Zg98, Vdeopt-MRV2, MRV2, MRV3, and F-RSV-MRV2. Tissue homogenates (1 g/mL) were infected with 6 log CCID_50_ of each virus and incubated at 37°C for 2 h. Infected tissues were centrifuged at 400 × *g* for 5 min at 24°C and washed 2 times with R10 medium to remove unbound virus. The infected suspensions were placed into 75-cm^2^ flasks, and 30 mL of medium was added prior to incubation (at 37°C and 5% CO_2_ for 10 days).

To assess the ability of MuV to infect and replicate in guinea pig tissues, tissue homogenate samples of lungs, brain, kidney, and testicles were infected with 12 different MuV strains (3 vaccine strains, 5 wild-type strains, and 4 recombinant viruses based on the L-Zagreb strain). High susceptibility of all analyzed tissue homogenates to infection with different MuV strains was found (Fig. 1). The achieved titers were in the range of 3.1 to 6.8 log cell culture 50% infective dose (CCID_50_)/mL in the lungs, 2.5 to 6.4 log CCID_50_/mL in the brain, 3.1 to 6.4 log CCID_50_/mL in the testes, and 2.3 to 7.3 log CCID_50_/mL in the kidneys.

### MuV infection of characterized guinea pig primary cell cultures.

**FIG 2:**
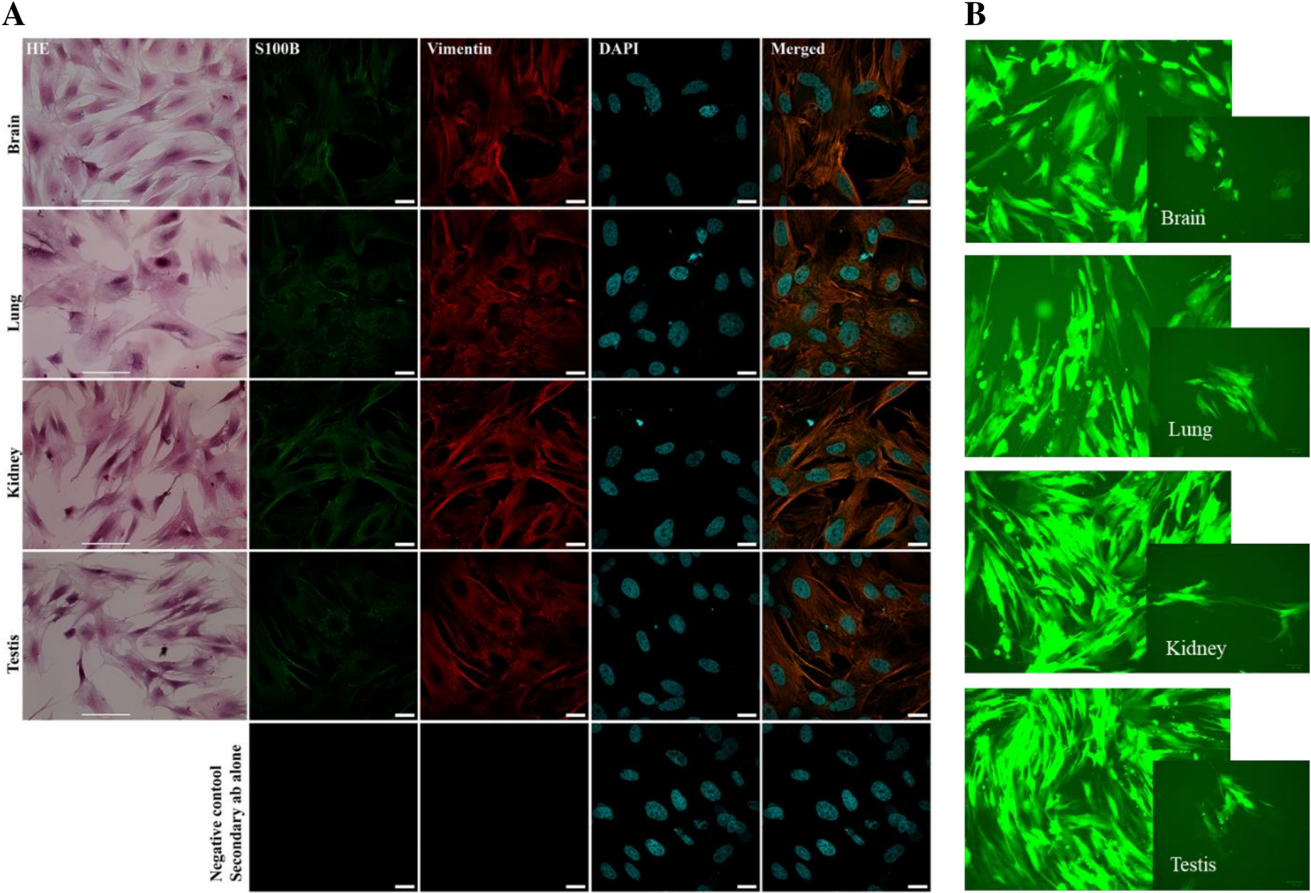
Characterization of primary cell cultures obtained from brain, lung, kidney, and testicles of guinea pig. (A) Uninfected guinea pig primary cell cultures. First column, the HE-stained cells show fibroblast-like morphology; second column, S100B (expressed in fibroblasts-positive cells); third column, vimentin (specific fibroblast marker-positive cells); fourth column, cells stained with DAPI; last row, negative control. (B) Guinea pig primary cell cultures 3 to 4 days following infection with MRV3 (MOI of 2).

Primary cell cultures of tissue homogenates from naive animals were cultivated, and adherent cells were subcultivated twice *in vitro*, using nondirecting, very basic cell culture medium. Microscopic inspection of cell morphology implied that mostly fibroblasts were cultivated, since the majority of cells were flat with big round to ellipsoid nuclei. Hematoxylin and eosin (HE) staining also showed fibroblast-like cell morphology (Fig. 2A). Moreover, immunostaining confirmed that primary cells, isolated from all four organs, were positive for fibroblast-specific markers (vimentin and S100B). In contrast, there were no cells positive for nestin (neural stem cell marker) or glial fibrillary acidic protein (GFAP) (astrocytes and glia-progenitor cell marker) (data not shown).

The same morphology was observed in the primary cell cultures of all four organs, after infection with MRV3 (recombinant MuV carrying a green fluorescent protein [GFP] gene which enables visualization of infected cells under a fluorescence microscope [Fig. 2B]). Within 3 to 4 days postinfection (dpi), all primary cells in the culture were infected. Round fluorescent formations visible in all infected cultures after 4 days were dead, infected cells (Fig. 2B).

These results demonstrate that all guinea pig primary cell cultures were highly susceptible to MuV infection.

### Expression of SAs at the surface of guinea pig primary cells.

**FIG 3:**
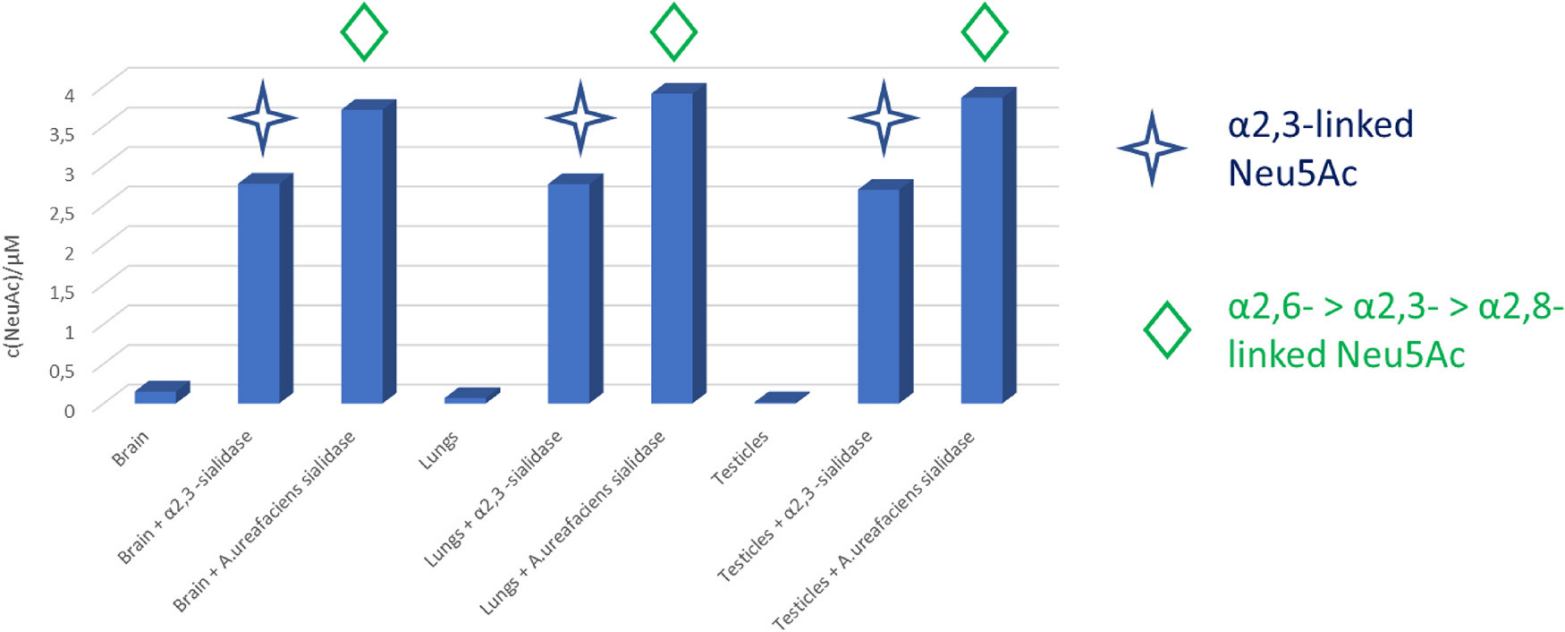
The amount of Neu5Ac released from the surface of guinea pig primary cells by treatment with A. ureafaciens (removes sialyl groups at rate α2,6→α2,3→α2,8-linkage) and α2,3-sialidase (removes α2,3-sialyl groups) followed by fluorescent labeling of SAs and gradient HPLC analysis.

**FIG 4:**
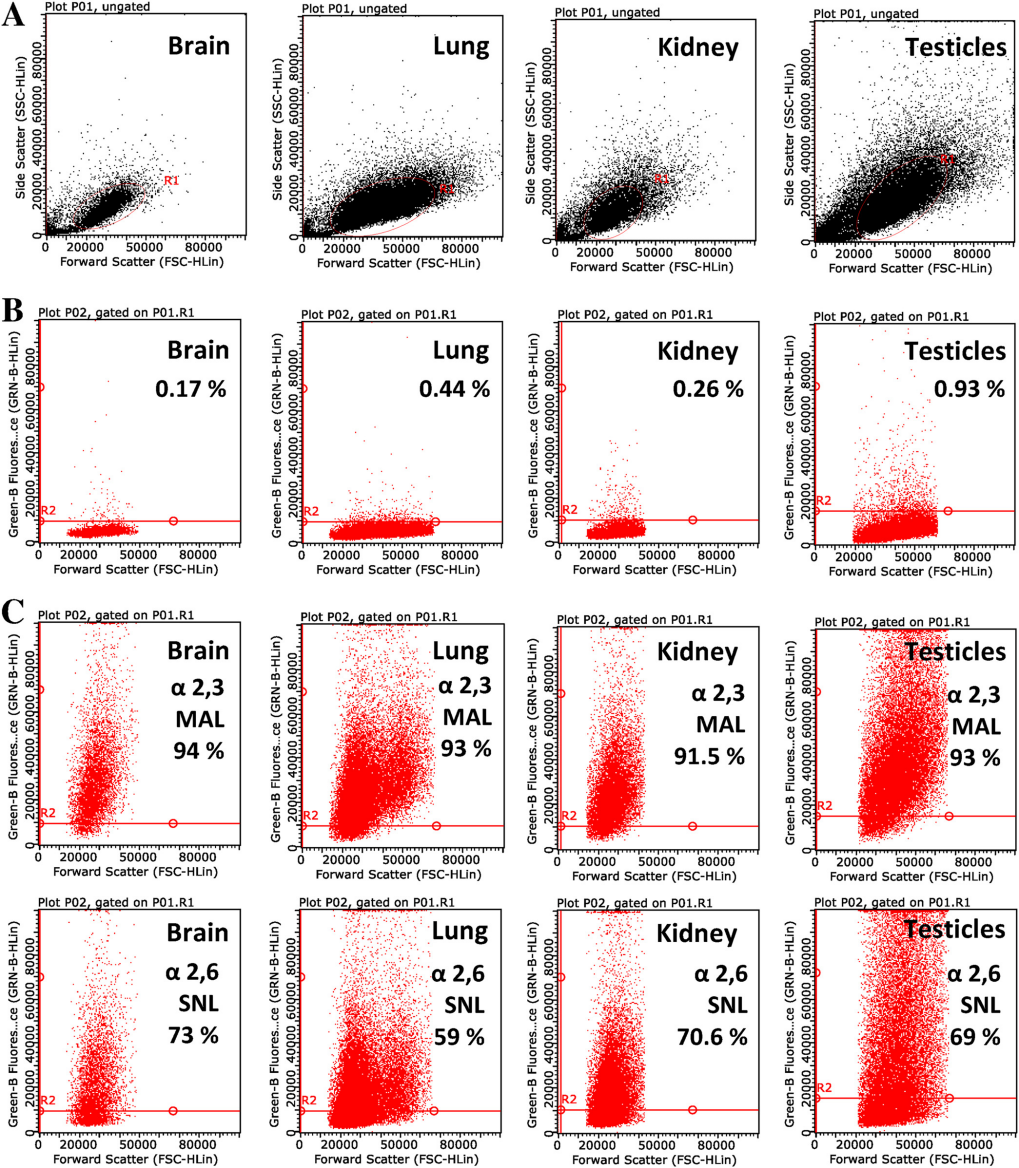
Determination of the SAs at the surface of primary cells obtained from different guinea pig organs by flow cytometry. (A) Untreated control cells; (B) cells stained with biotinylated MAL; (C) cells stained with biotinylated SNA lectin (SNL).

Since it was shown that a trisaccharide containing α2,3-linked sialic acid at the cell surface acts as a receptor for MuV (2, 3), the expression of SAs at the surface of guinea pig primary cell cultures from brain, testicles, lung, and kidney was measured. SAs were released by treatment with α2,3-sialidase (removes α2,3-sialyl groups) and Arthrobacter ureafaciens sialidase (removes the following sialyl groups at rate α2,6→α2,3→α2,8-linkage). The latter treatment released more *N*-acetylneuraminic acid (Neu5Ac) than α2,3-sialidase in all tested samples (Fig. 3). If sialidase treatments for 2 h released all susceptible surface Neu5Ac, then A. ureafaciens sialidase released both α2,3- and α2,6-linked Neu5Ac, while α2,3-sialidase released only α2,3-linked Neu5Ac. Therefore, by comparison of released Neu5Ac with both sialidases, it can be assumed that in all analyzed cells α2,3-linked Neu5Ac contains about 70% of total Neu5Ac present at the cell surfaces. The presence of both α2,3-linked and α2,6-linked SAs, with α2,3-linked SAs being more abundant, was additionally confirmed by flow cytometry analysis (Fig. 4).

### Experimental i.n. and i.t. infection of guinea pigs with MuV.

After observing that different MuV strains replicate well in different guinea pig tissue homogenates and that cells from different tissues possess α2,3-linked SAs on their surfaces, we experimentally infected guinea pigs to examine if MuV replication could be detected *in vivo.* One group of animals was i.n. infected with MRV3 (1 × 10^6^ PFU) (to mimic the natural route of MuV infection), and another group of animals was i.t. infected with the same virus dose (as an iatrogenic route of infection of the tissue that is naturally susceptible to MuV infection).

**FIG 5:**
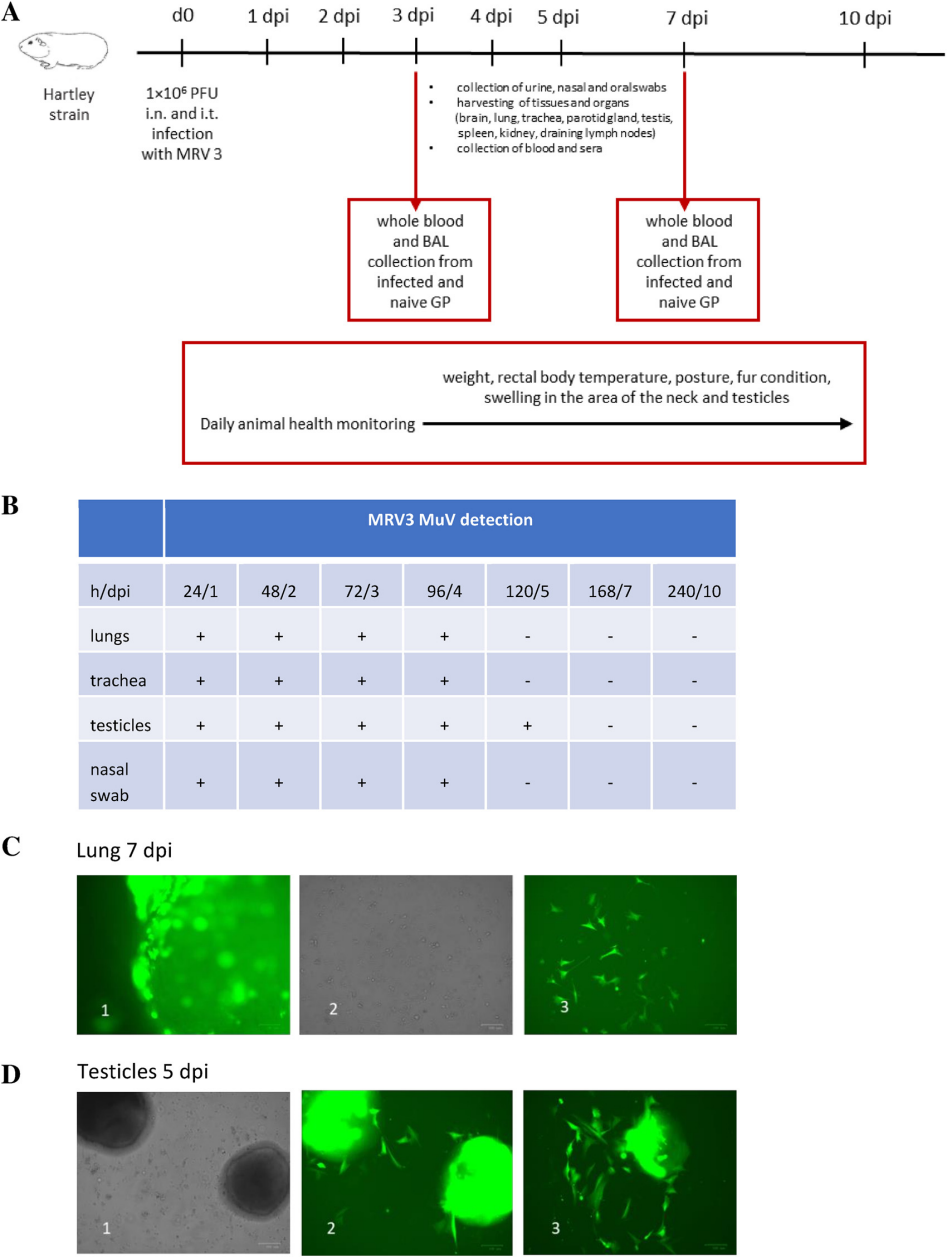
Experimental i.n. and i.t. infection of guinea pigs (GP) with MRV3. (A) Scheme of experimental i.n. and i.t. infection of guinea pigs with MRV3. (B) Evidence of virus replication in infected lungs, trachea, testicles, and nasal swab depending on the time postinfection. (C) Tissue homogenate of infected lung at 7 dpi: (1) infected lung tissue (there were no proliferated cells in the area); (2) lung cell images without fluorescent labels; (3) lung cell images with fluorescent labels. (D) Tissue homogenate of infected testicles at 5 dpi: (1) testis cell images without fluorescent labels; (2) testis cell images with fluorescent labels; (3) virus spread from infected tissue to surrounding cells.

Infected guinea pigs exhibited no clinical signs of disease such as weight loss, fever, or swelling of testes, head, or neck throughout the experiment. On days 1, 2, 3, 4, 5, 7, and 10 after infection, animals were euthanized and different tissues, organs, and samples were collected (brain, lung, trachea, parotid gland, testis, spleen, kidney, draining lymph nodes, urine, feces, sera, and nasal and oral swabs) for virus detection (Fig. 5A).

Tissue homogenates were prepared and cultured as described previously to determine if a replicative virus was present in the culture of infected tissue (*ex vivo* monitoring). After the incubation period of 10 days, the virus was detected only in the culture of lungs and trachea obtained from i.n.-infected animals and in the testis tissue homogenates originating from i.t.-infected animals (Fig. 5B). Viral shedding to any other tissue or organ was not detected. The virus could be isolated from the lungs and trachea for up to 4 dpi and from the testicles for up to 5 dpi.

The replication-competent virus was also isolated from the nasal swabs of all tested animals and from only several mouth swab samples. On the other hand, virus could not be isolated from the urine, feces, sera, or draining lymph nodes.

These results indicate that guinea pigs are susceptible to MuV infection which is asymptomatic. I.n.-inoculated MuV reaches through the trachea to the guinea pig lungs, where it replicates to a limited extent, and it is cleared within 4 to 5 dpi.

In addition, to address potential virus transmission through direct contact among animals, each of four groups of guinea pigs i.n.-infected the previous day was cohoused with one naive guinea pig. Oral and nasal swabs from naive guinea pigs were collected every day from days 1 to 7 during cocaging with infected animals. Despite the high viral pressure, no direct contact transmission of MuV among guinea pigs was observed.

### Activation of immune responses in guinea pigs infected with MuV.

To obtain more insight into immune responses mounted in infected animals, first a flow cytometry analysis was used to address potential changes in the composition of the leukocyte cell populations from the whole blood or bronchoalveolar lavage fluid (BALF). Two test points were chosen: 3 dpi, when the presence of the virus was demonstrated in the lungs and trachea, and 7 dpi, when replication-competent virus was no longer detected.

**FIG 6:**
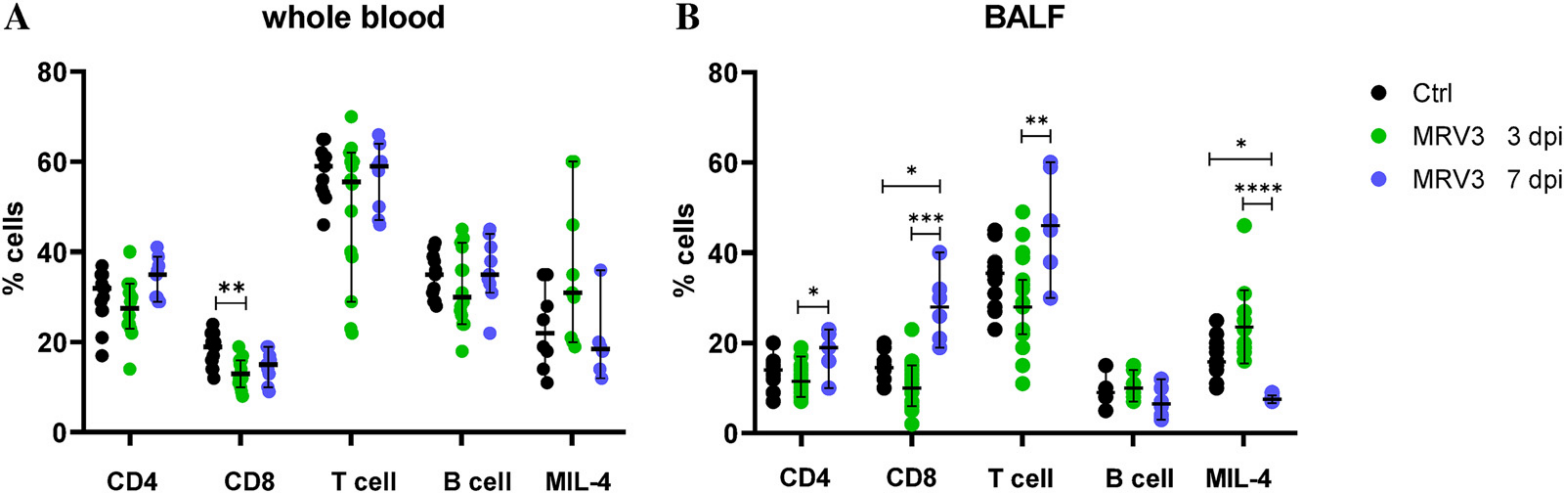
Flow cytometry analysis of lymphocyte and leukocyte subsets in MuV-infected guinea pigs on days 3 and 7 postinfection in whole blood (A) and BALF (B). Results for each group are shown as median with 95% confidence interval (*n* = 6 to 17).

On day 3 postinfection (p.i.), significantly reduced numbers of CD8^+^ T cells (*P* = 0.0067) were found in the whole blood of infected animals compared to the control animals (Fig. 6A). On the other hand, the bronchoalveolar immune response in MuV-infected guinea pigs showed a substantially different immune profile; the percentage of CD8^+^ and total T cells significantly increased (*P* = 0.0002 and *P* = 0.0063, respectively) from day 3 to day 7 p.i. (Fig. 6B). The CD4^+^ T cells were also increased (*P* = 0.0328) in infected compared to control animals. Additionally, significant change in the number of MIL4 positive cells (granulocytes and monocytes/macrophages) was recorded in infected animals; after a slight increase on day 3 p.i., there was a strong decrease on day 7 p.i. (*P* < 0.0001). There were no changes in the number of B cells in infected animals compared to controls.

**FIG 7:**
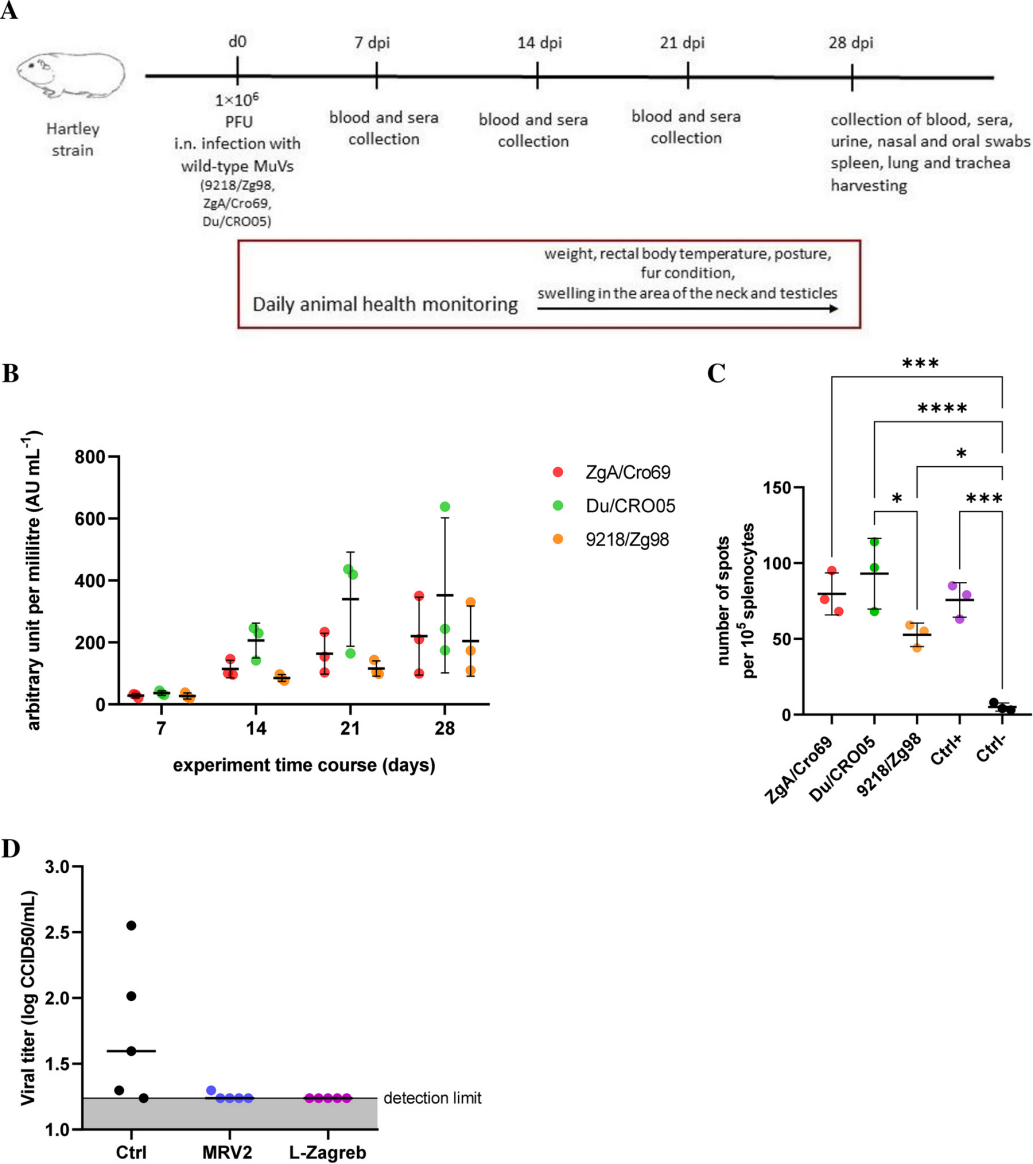
Humoral and cellular responses in infected guinea pigs. (A) Schematic diagram of infection protocol. Guinea pigs were infected with 1 × 10^6^ PFU of 3 MuV wild-type strains (ZgA/Cro69, Du/CRO05, and 9218/Zg98) or medium (control group) via the intranasal route. Blood samples (whole blood and sera) were collected on days 7, 14, 21, and 28 postinfection. (B) MuV-specific antibody response against different strains was measured by ELISA. Mean values ± standard deviations (*n* = 3) are shown. (C) The induced T-cell (IFN-γ) response was measured by ELISpot assay. Mean values ± standard deviations (*n* = 3) are shown. (D) MuV challenge assay. Unvaccinated control animals and animals s.c. vaccinated with 1 × 10^6^ PFU of L-Zagreb vaccine strain or recombinant MuV (MRV2) were i.n. challenged with 1 × 10^7^ PFU of MuV MRV3. The presence of infective virus in BALF from each animal was determined 3 days later by CCID_50_ assay. The median value for each animal group (*n* = 5) is shown by a solid line.

To further investigate the capability of guinea pigs to mount a MuV-specific adaptive immune response, 3 guinea pigs per group were i.n. infected with 1 × 10^6^ PFU of each of the 3 wild-type MuV strains (ZgA/Cro69, Du/CRO05, and 9218/Zg98) (Fig. 7A). Three control animals received saline only. During the entire infection protocol (28 days), guinea pigs did not display any obvious signs of illness, such as weight loss, fever, or behavioral changes. Blood samples were collected on days 7, 14, 21, and 28 p.i., and antibody titers against MuV were measured by enzyme-linked immunosorbent assay (ELISA). As shown in Fig. 7B, all wild-type MuV strains were able to induce antibody responses in guinea pigs. Throughout the follow-up period, a significant increase in mumps-specific IgG antibodies was observed up to day 28 postinfection. The largest increase in antibody titer was caused by strain Du/CRO05, followed by ZgA/Cro69, while the smallest increase was observed for strain 9218/Zg98 (Fig. 7B). In addition, we could not detect any changes in the complement activity in samples obtained from infected animals compared to controls (data not shown).

Splenocytes were collected in the terminal test point and analyzed for T-cell responses by performing the enzyme-linked immunosorbent spot (ELISpot) assay to measure IFN-γ production. Splenocytes from 4 treatment groups (control group and groups infected with ZgA/Cro69, Du/CRO05, or 9218/Zg98) were stimulated *in vitro* with the respective viruses (multiplicity of infection [MOI] of 2). All wild-type MuV strains induced detectable cellular immune responses (Fig. 7C).

Efficacy of immune response was assessed by measuring the presence of an infectious virus in BALF on the 3rd day after MuV challenge of previously vaccinated guinea pigs. Guinea pigs (5 per group) were subcutaneously (s.c.) immunized (two times with 1 × 10^6^ PFU per animal in a 3-week interval) and then challenged with MRV3 at a 10-fold-higher dose (1 × 10^7^ PFU per animal) 2 weeks later. In the group of guinea pigs immunized with the recombinant virus (MRV2), only one animal after the challenge showed the presence of the virus in a low titer (Fig. 7D). In the group of guinea pigs immunized with the vaccine strain (L-Zagreb), no infectious virus was detected in any animal. On the other hand, in the control group of animals, the infectious virus was detected in all BALF samples except for one (Fig. 7D).

The obtained results clearly show that animals i.n. infected with MuV develop detectable antibody titers and cellular immune responses during 28 days. Also, animals immunized with the mumps virus develop a protective immune response that effectively shields them from challenge with the live virus.

### Histopathological analysis of the lungs and testicles from MuV-infected guinea pigs.

**FIG 8:**
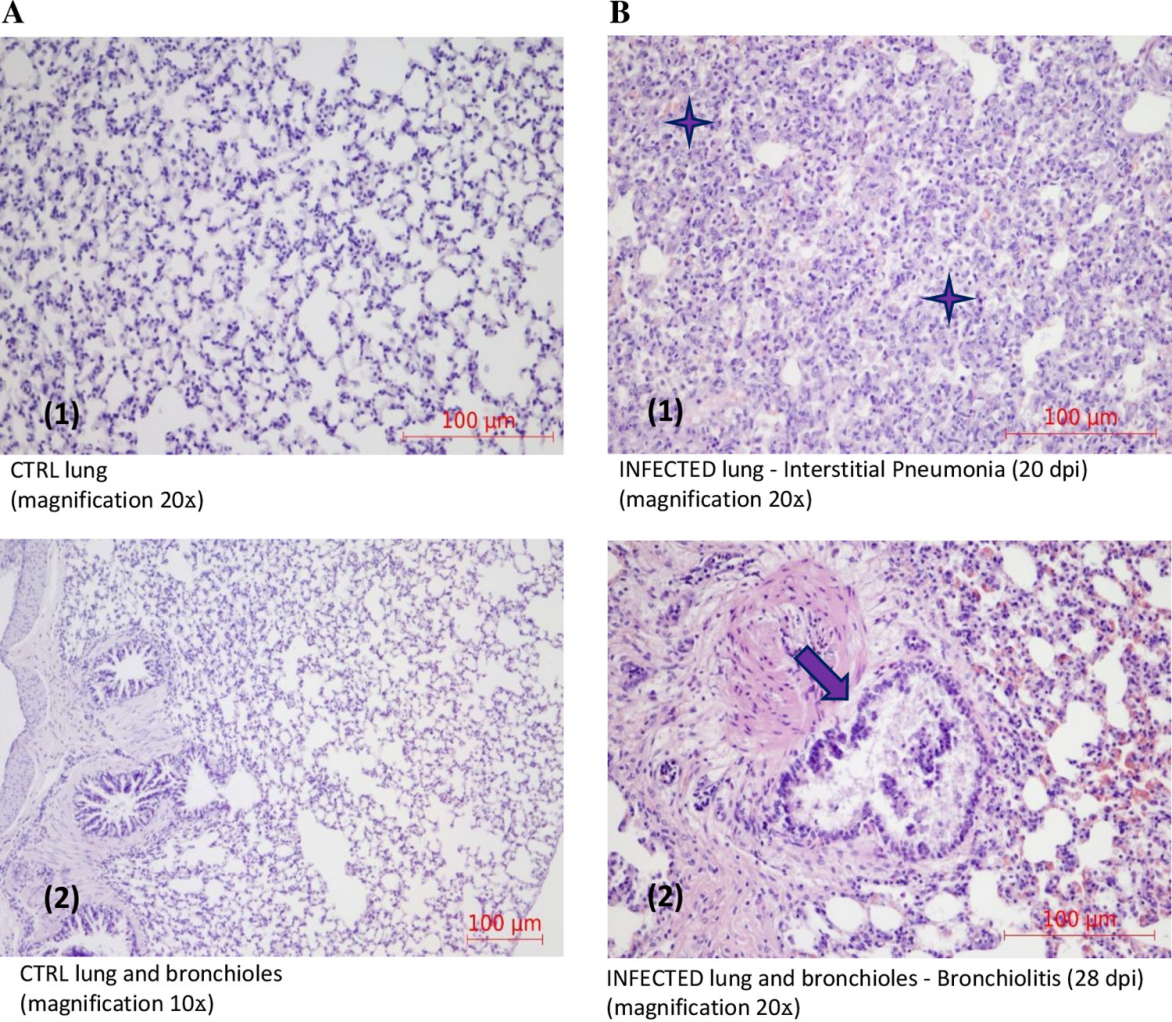
Histopathological findings of MuV activity in guinea pig lungs. (A) Lung of control guinea pig shows thin interalveolar septa with clear intra-alveolar spaces (1) and bronchioles lined with columnar epithelium and empty lumen (2). (B) Lung of MuV-infected guinea pig shows interstitial pneumonia (marked with asterisks) with thickened interalveolar septa with subsequent reduction of alveolar spaces (1). The bronchioles are lined with an attenuated epithelium that exfoliates into the lumen, where it is admixed with mucinous material (marked with arrow) (2).

Histopathological examination of the lungs of i.n.-infected guinea pigs at the terminal test point (28 dpi) showed that all lung lobes were affected by inflammatory changes (Fig. 8). In all infected animals, similar lesions were found in the trachea and lungs, regardless of the strain used. Lungs were affected with multifocal to confluent interstitial pneumonia characterized by thickened interalveolar septa due to mild pneumocyte hypertrophy and moderate, rarely severe infiltration of lymphocytes, few plasma cells, and heterophils with subsequent reduction of alveolar spaces. Occasionally, mild mucinous and heterophilic bronchiolitis was noticed. Interstitial pneumonia was of slightly higher intensity in groups infected with ZgA/Cro69 and Du/CRO05 than with 9218/Zg98.

**FIG 9:**
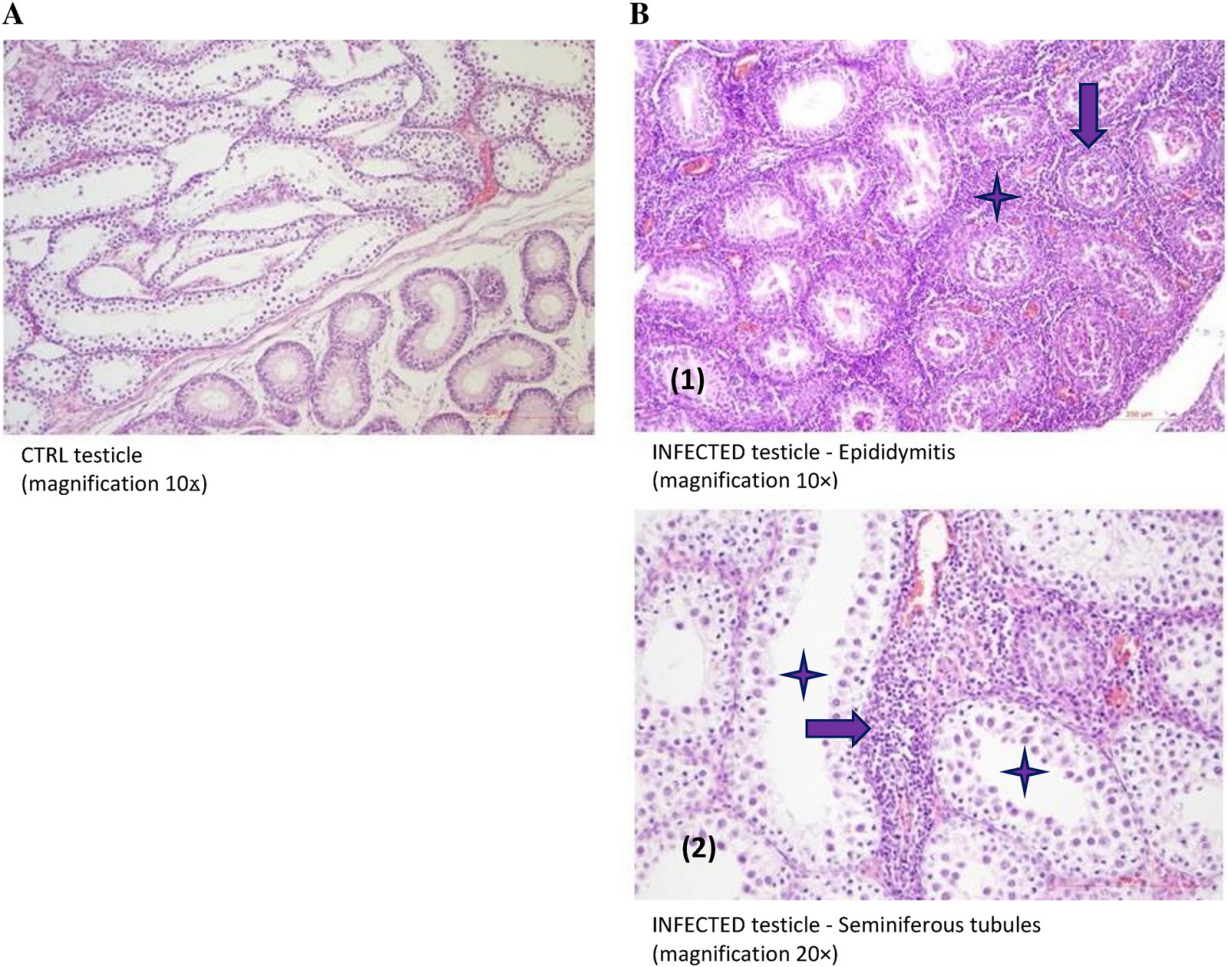
Histopathological findings of MuV activity in guinea pig testicles. (A) Testicle of control guinea pig. (B) Testicle of MuV-infected guinea pig showing mild to moderate necrotizing and lymphocytic epididymitis; the tubules of the epididymis are filled with necrotic cellular debris (marked with arrow), and a lymphocytic infiltrate is visible in the interstitium (marked with asterisk). Spermatozoa are not visible (1). Testicle of MuV-infected guinea pig showing seminiferous tubules lined mostly with Sertoli cells, spermatogonia, and rare spermatocytes (marked with asterisks). In the interstitium, predominantly perivascular, there is a lymphocytic infiltrate (marked with arrow) (2).

Histopathological examination of the testicles of the i.t.-infected guinea pigs at 7 dpi showed that testicular lesions were characterized by mild, predominantly lymphocytic interstitial orchitis (Fig. 9). The seminiferous tubules were lined predominantly with Sertoli cells and spermatogonia with rare spermatocytes, while other developmental stages of germ cells were missing. In the epididymis there was mild to moderate necrotizing and lymphocytic epididymitis (Fig. 9).

## DISCUSSION

Mumps is a highly contagious disease which spreads through direct contact with saliva or respiratory droplets from the mouth, nose, or throat. After more than a half-century of declining mumps incidence due to the widespread use of vaccines, even populations with good vaccination coverage are again experiencing small or large outbreaks of mumps (11, 20). It is not entirely clear whether immune protection is weakening or whether existing vaccines inadequately protect against new circulating MuV strains. The scientific community is faced with a lack of a good animal model for testing MuV pathogenesis as well as the existing or new mumps vaccines and their efficacy.

The sensitivity of guinea pigs to infections and the similarities of their immune system to that of humans have made this animal model important in the study of human infectious diseases (21–23). Between the 1950s and 1990s, the guinea pig was used as a model for MuV research. However, to date, only a few studies have been conducted in which the interaction between guinea pig and MuV has been studied to a very limited extent. In those experiments, animals were infected with MuV intraocularly or intracerebrally or in the animal’s ear (24–27). Unfortunately, none of the routes of infection in the mentioned studies are relevant to the natural route of infection. Furthermore, the only immunological parameter that was investigated was the humoral immune response. However, with the development of new generations of transgenic mouse strains that are much less expensive to maintain as well as the lack of established procedures and reagents for comprehensive immunological analysis on the guinea pig, the guinea pig as a model for MuV research has been almost abandoned (28, 29). The only promising model for evaluating mumps immunity in mice was type I interferon (IFN) alpha/beta receptor knockout mice (29) deficient for normal immune response.

More recently, researchers have willingly returned to guinea pigs as a model for mumps vaccine research, despite its robustness to severe infection that would include the onset of symptoms (30). The guinea pig is the choice for research on immunogenicity of MuV or new mumps vaccine candidates (30–33). However, in all of those studies the only immunological parameter that was investigated was the humoral immune response. Development of new tools for detection of humoral and cellular immune responses such as guinea pig-specific IFN-γ ELISpot assay (34) or improving antibody selection for flow cytometry analysis (23) provided a better insight into the development of cellular immunity in guinea pigs during experimental MuV infection, as well as long-term immunity. For all the above reasons, in this study we decided to examine the guinea pig and its interaction with MuV in more detail.

First, we demonstrated the high susceptibility to MuV infection for different guinea pig tissue homogenates (brain, lung, kidney, and testis), as well as their primary cell cultures, regardless of the strain used. Several different recombinant viruses were used in this study to investigate whether targeted changes in the genome (introduction of foreign genes or modification of existing genes) can have an effect on the infectivity and replicative capacity of MuV in guinea pig cells. MRV3 virus was used to unequivocally visualize and confirm the presence and replication of the virus in tissues and primary cell cultures. In addition, we wanted to see if the different origins of the viruses with the same consensus sequence (L-Zagreb produced in the traditional way by passaging in chicken fibroblasts and MRV2 prepared by the rescue process) have an effect on the infectivity of the virus in guinea pig cells. Despite the changes they have in the genome, the recombinant viruses infected the primary cells of guinea pigs with the same degree of ubiquity and, like the original strain L-Zagreb, successfully replicated.

In the history of mumps research, cells originating from guinea pigs have been used. Gavitt and Bird (26) showed that MuV replicates in guinea pig heart cells. Two MuV strains (Sofia 6 and Leningrad-3) were prepared in primary cultures of guinea pig kidney cells and used as vaccine strains in Bulgaria and Russia in the middle of the last century (35). Also, Parker in his work (36) used a continuous cell line of guinea pig lungs for MuV infection (JH4 Clon 1 fibroblast-like cells) but did not obtain a titer higher than 3.3 log PFU/mL.

Our analysis showed the presence of both α2,3-linked and α2,6-linked SAs at the surface of guinea pig primary cells of all analyzed organs (brain, lungs, kidney, and testes), with α2,3-linked SAs being more abundant. The same finding was obtained by Sun et al. (37), but only on respiratory tissue. In our study, the amounts of SAs in the samples were determined by comparing the response in the sample to a curve from Neu5Ac and *N*-glycolylneuraminic acid (Neu5Gc) standards. In all tested samples, high levels of Neu5Ac and only low concentrations of Neu5Gc were found (see Fig. S1 in the supplemental material). Possible causes of this finding are that the sialidases used generally have higher affinity for Neu5Ac than for Neu5Gc residues or that only a small amount of Neu5Gc is present at the surface of tested guinea pig primary cells (38). Our data show that, due to ubiquitous surface expression of α2,3-linked SAs, guinea pig cells meet a main prerequisite for successful MuV cell entry and spread.

Furthermore, we have confirmed that guinea pig tissue homogenates grown in cell culture basic medium yield primary cultures mostly made of fibroblasts. It is known that fibroblasts are a heterogeneous population of cells (5, 39) with specific roles depending on their tissue origin. The high affinity of MuV toward fibroblast cells is interesting The high affinity of MuV for fibroblast cells is interesting because of the role of fibroblasts in the inflammatory processes (39). On the other hand, based on the current knowledge of MuV pathogenesis, epithelial cells are the primary targets of MuV in humans (5). This finding opens up space for investigating the susceptibility of other tissue- or organ-specific cells to MuV, as well as the potential for the development of an *in vitro* model for examining the cellular pathogenesis of MuV in mixed cell cultures.

To investigate MuV pathogenesis in guinea pig, we infected guinea pigs in 2 ways, intranasally and intratesticularly. All *in vivo* experiments were performed using MRV3 (fluorescently labeled MuV) at a dose of 1 × 10^6^ PFU, allowing us to more easily monitor virus replication. Within the first 7 days of infection, we monitored the retention of the virus in the infected organs, the development of the clinical picture, and different parameters of cellular and humoral immunity. Guinea pigs exhibited no clinical signs of disease, but live MuV was recovered from nasal swab or tissue homogenates of testes, trachea, and lung up to 5 dpi. In contrast to the previous finding by Tokuda (25), our data show that i.t.-inoculated virus persists in the testes for more than 3 days. Also, MRV3 was detected in nasal swabs of infected animals up to 5 days. Despite this, we were unable to demonstrate MuV transmission from infected to naive guinea pigs in a cocaging experiment, as demonstrated for influenza virus infection (40, 41).

Dynamic changes in the guinea pig cellular immune responses to MuV infection were observed on day 7 p.i., but only in BALF. An increase in CD8^+^ T cells and total T lymphocytes indicated an acute viral infection. CD8^+^ T cells are critical for mediating clearance following many acute viral infections in the lungs (42, 43). Furthermore, a slight increase in MIL4 positive cells, mainly neutrophils and monocytes/macrophages (44), in BALF on day 3 p.i. suggests an influx of granulocytes into infected organs (45) and thereby supports active infection of the guinea pigs’ respiratory system following i.n. infection. Together, these data suggest that MuV infection activated cellular immunity mechanisms within the first 7 dpi.

In the experimental infection of guinea pigs with 3 wild-type MuV strains (ZgA/Cro69, Du/CRO05, and 9218/Zg98), we also demonstrated that animals develop detectable humoral response after only one encounter with MuV at a dose of 1 × 10^6^ PFU. All guinea pigs showed a clear increase in mumps-specific antibody titer between days 21 and 28 p.i.

The observation of increased levels of IFN-γ in spleen and activation of T lymphocytes in BALF and whole blood is consistent with the viral infection (46) and suggests they had been infected. Activated CD8^+^ T cells are also known to acquire effector functions after viral infection (42, 47). Virus-specific CD8^+^ T cells rapidly produce cytokines, including IFN-γ and tumor necrosis factor (TNF), that can directly or indirectly induce cell death of virus-infected cells (42).

To confirm the efficiency of preexisting immunity, a challenge test was performed in immunized guinea pigs that clearly showed the absence of virus in BALF of immunized animals. This also confirmed the potential of guinea pigs as a model for vaccine development and testing.

Finally, the pathological changes in infected organs (moderate to severe pneumonia and interstitial orchitis) are indisputable evidence of MuV infection and replication, which is the first such evidence in guinea pigs.

An important component of the innate immune response to viruses is the complement system, which has the ability to recognize and eliminate viruses (48). We, therefore, wanted to investigate whether complement is involved in the acute MuV infection in guinea pig tissues. However, analysis of the total complement did not reveal any changes in its activity.

Taken together, our results demonstrate that (i) guinea pigs can be infected *in vivo* with MuV after intranasal inoculation, (ii) infectious MuV can be detected in lungs 4 days after infection, and (iii) it induces both cellular and humoral immune response in infected animals. A finding that MuV replicates and induces pathology in the animal’s organs (lung and testicles) and replicates in the primary cell cultures of different animal tissues relevant to the pathology of this virus in humans presents the basis for using this model in further investigation of largely unexplained MuV pathogenesis. Induction of both cellular and humoral immune responses after the natural route of infection also gives the possibility for better characterization of the immune response induced by immunization or natural MuV infection.

## MATERIALS AND METHODS

### Cells and viruses.

Vero cells (African green monkey kidney cells; European Collection of Animal Cell Cultures [ECACC]) were maintained in culture using minimal essential medium with Hanks’ salts (MEM-H) (Capricorn Scientific, Ebsdorfergrund, Germany) supplemented with 10% fetal bovine serum (FBS) (PAN-Biotech, Aidenbach, Germany) and penicillin/streptomycin and l-glutamine (both from Capricorn Scientific, Ebsdorfergrund, Germany) at 37°C and 5% CO_2_.

MuV strains used in this study included: ZgA/Cro69 (genotype D) (49), ZgB/Cro69 (unclassified) (49), 9218/Zg98 (genotype C) (50), L-Zagreb (genotype N) (Institute of Immunology, Inc.), Urabe AM9 (genotype B) (1st International Reference Reagent for Mumps Vaccine, National Institute for Biological Standards and Control [NIBSC]), JL2 (genotype A) (a kind gift from B. K. Rima), Du/CRO05 (genotype G) (50), and MuVi/Split.CRO/05.11 (genotype G) (isolated in Croatia in 2011). Recombinant viruses MRV2, Vdeopt-MRV2, F-RSV-MRV2, and MRV3 (NCBI GenBank accession numbers MZ929423, MZ964864, MZ964861, and MZ929424, respectively) were based on the L-Zagreb strain with several modifications and generated as described in the work of Slović et al. (51).

All viruses were propagated in Vero cells for up to 3 passages. Viruses were grown in MEM-H containing 5% FBS, penicillin/streptomycin, and l-glutamine at 35°C and 5% CO_2_ until the cytopathic effect (CPE) was observed. Virus titer was determined by CCID_50_ or plaque assay (PFU) according to the work of Forčić et al. (52).

### Animals.

Animal experimentation was approved by the Croatian Ministry of Agriculture, Veterinary and Food Safety Directorate (UP/I-322-01/17-01/74, permission no. 525-10/1315-20-11, date 29 April 2020) and performed in accordance with the Croatian Law on Animal Welfare, which complies with EU Directive 2010/63/EU. Hartley guinea pigs of both sexes, 4 to 6 weeks of age, weighing 300 to 500 g, were provided by the Institute of Immunology Inc., Croatia. The animals were housed in groups in standard cages (4 per cage), in air-conditioned rooms, at 22 ± 3°C and 55% ± 5% humidity with 12-h light and 12-h dark cycles. For the entire period of each experiment, animals had access to standard diet for guinea pigs (Muchedola Srl., Italy) and water *ad libitum* and received daily a hay and environmental enrichment (Tapvei, Estonia).

### Preparation of tissue homogenates and cultivation of guinea pig primary cell cultures.

The guinea pigs were deeply anesthetized by an intraperitoneal injection of ketamine (60 mg/kg of body weight) (Ketamidor 10% solution; Richter Pharma, Austria) and xylazine (5 to 10 mg/kg) (Xylased bio, 20 mg/mL; Bioveta a.s., Czech Republic) and then euthanized with euthanasia solution (Euthasol, 400 mg/mL; Genera, Croatia). The brain, lung, kidney, and testicles were removed immediately and washed in sterile phosphate-buffered saline (PBS, pH 7.3 to 7.4) (Capricorn Scientific, Ebsdorfergrund, Germany) containing 1% penicillin and streptomycin. The tissue processing was carried out in a biosafety cabinet. Each organ was cut into small pieces (1 mm^2^) and homogenized. Tissue homogenates were centrifuged and cells were resuspended in 3 to 5 mL R10 medium (Capricorn Scientific, Germany) supplemented with 10% FBS, 1% penicillin and streptomycin, and 1% l-glutamine. Depending on the organ, 500 to 1,000 μL of tissue homogenate was seeded in a 150-cm^2^ flask and cultured at 37°C with 5% CO_2_ and saturated humidity. When primary cells reached 80 to 90% confluence, the cell culture was dissociated using 0.25% trypsin and further grown for up to 2 additional passages. Tissue homogenates of infected animals were prepared in the same manner.

### Characterization of guinea pig primary cell cultures.

Hematoxylin and eosin (HE) staining was performed using a commercially available kit (HEMM-OT-100, EOY-05-OT-1L; Biognost) according to the manufacturer’s instructions. Immunostaining was performed according to the work of Alić et al. (53). Briefly, cells were fixed with 4% paraformaldehyde solution and rinsed with PBS. Permeabilization and blocking were carried out in 3% donkey serum with 0.2% Triton X-100 in PBS for 1 h at room temperature (RT). The cells were incubated with primary antibody diluted in 1% donkey serum with 0.2% Triton X-100 in PBS overnight at 4°C. The cells were washed with PBS and incubated with secondary antibody solution for 2 h at RT. Cells were rinsed with PBS and counterstained with 4′,6-diamidino-2-phenylindole (DAPI) for 10 min at RT and then rinsed three times with PBS and mounted with Dako fluorescent mounting medium. As a negative control for all antibody combinations, incubation with secondary antibody alone was carried out. Lists of primary and secondary antibodies used in the study are shown in Table S1 and Table S2 in the supplemental material, respectively. Stained coverslips were analyzed using an EVOS FL Auto System (Thermo Fisher Scientific), a fluorescence microscope (HE staining), and an Olympus FV 3000 confocal microscope.

### Infection of guinea pig tissue homogenates with MuV strains.

Suspensions of tissue homogenates (lung, brain, kidney, and testicles) were infected separately with 12 previously described MuV strains. Tissue homogenates (1 g/mL) were infected with 6 log CCID_50_ of each virus and incubated at 37°C for 2 h. Infected tissues were centrifuged at 400 × *g* for 5 min at 24°C and washed 2 times with R10 medium to remove the unbound virus. The infected suspensions were placed into 75-cm^2^ flasks, and 30 mL of medium was added prior to incubation (at 37°C and 5% CO_2_ for 10 days). On day 10, supernatant aliquots were removed, centrifuged, and stored at −80°C until virus titration by CCID_50_ assay.

### Removal of SAs from the surface of guinea pig primary cells.

The first passage of primary cell cultures originating from different guinea pig tissues was seeded and cultured in 6-well plates (1.0 × 10^6^ cells/well) in R10 medium for 24 h. The sialidase cloned from A. ureafaciens (Merck, USA) was added to the culture medium at 20 mU/mL to remove both α2,3- and α2,6-linked SAs on glycoproteins and glycolipids. Then, an α2,3-sialidase cloned from Salmonella enterica serovar Typhimurium LT2 (TaKaRa Bio, USA) was added to the culture medium at 200 mU/mL to remove α2,3-linked SAs. The treated cells were incubated at 37°C for 2 h. The supernatants were aspirated and centrifuged for 10 min at 2,000 × *g* for high-performance liquid chromatography (HPLC) analysis.

### Fluorescent labeling of SAs and HPLC analysis.

Released SAs from primary cell cultures and SA standards (Neu5Ac and Neu5Gc, obtained from Tokyo Chemical Industry, Tokyo, Japan) were fluorescently labeled with 1,2-diamino-4,5-methylenedioxybenzene dihydrochloride (DMB) (Sigma-Aldrich, St. Louis, MO, USA) at 50°C for 3 h in the dark (54).

The procedure was performed with certain modifications according to the product guide for the LudgerTag DMB sialic acid release and labeling kit (54). Freshly prepared DMB labeling reagent (20 μL) was added to each sample of free SAs (25 μL of sample and 5 μL of 8.4 M acetic acid) and to each SA standard (2.5 μL of 0.5 mM Neu5Ac or Neu5Gc and 2.5 μL of 2.8 M acetic acid). The tubes with samples and standards were mixed using a vortex mixer and heated in a thermomixer at 50°C for 3 h in the dark. The reaction of fluorescence labeling of SAs was terminated by adding 450 μL of water to each sample and 600 μL of water to each SA standard. After termination, the tubes with samples and standards were centrifuged at 3,000 × *g* for 5 min. Standards were diluted for standard curves. Six standard solutions were obtained by appropriate dilution of 2 μM Neu5Ac or Neu5Gc with water in the concentration range of 2 to 0.025 μM Neu5Ac and 1 to 0.004 μM Neu5Gc. Samples and standards were transferred to vials for HPLC analysis.

The quantification of DMB-labeled SAs was determined by a gradient HPLC method with fluorescence detection after optimizing the conditions for separation and quantification of SAs in tested samples (Fig. S1 and S2). Briefly, HPLC analyses were carried out using the Shimadzu Prominence LC-20AD with an RF-10A XS fluorescence detector, a SIL20AC HT autosampler, and LabSolutions software (Kyoto, Japan). The chromatographic separation was performed on the LudgerSep R1 HPLC column, 4.6 by 150 mm, 3 μm (Abingdon, UK), with a 2-SAV5 in-line filter (Neston, UK). The mobile phase consisted of a mixture of acetonitrile-methanol-water at 9:7:84 (A) and acetonitrile (B). The percentage of acetonitrile at 0, 19, 19.5, 23.5, 24, 30, and 37 min was 0, 0, 90, 90, 0, 0, and 0, respectively. The injection volume was 25 μL. Analyses were run at a flow rate of 0.5 mL/min at RT, and eluates were monitored using a fluorescence detector. A fluorescence detector was set at 373 nm for excitation and 448 nm for emission.

### Analysis of surface SAs by lectin staining.

Surface SAs were measured using the biotinylated Maackia amurensis lectin II (MAL II) specific for binding to α2,3-linked SAs and biotinylated Sambucus nigra (elderberry) bark lectin (SNA, EBL), which preferentially binds to SAs attached to the terminal galactose via α2,6 - linkage. Lectins were purchased from Vector Laboratories (Burlingame, CA, USA). Primary cultures of guinea pig cells (lung, testes, and brain) were washed with PBS, detached using 0.25% trypsin-EDTA, and washed once in R10 medium and twice with 1% bovine serum albumin (BSA)-PBS. The cells (1 × 10^6^ per 100 μL) were divided into three 1.5-mL Eppendorf tubes and incubated with 1% BSA-PBS for 30 min at RT. These aliquots were incubated with biotinylated MAL II (10 μg/mL 1% BSA-PBS with Ca^2+^), biotinylated SNA (10 μg/mL 1% BSA-PBS with Ca^2+^), or the lectin-free medium (1% BSA-PBS) for 30 min at 4°C with gentle shaking. The cells were washed and incubated with fluorescein isothiocyanate (FITC)-avidin (Ultra avidin fluorescein; Leinco Technologies) in 1% BSA-PBS for 30 min at 4°C with occasional gentle shaking. After washing with 1% BSA-PBS, the cells were analyzed on a Guava easyCyte flow cytometer (Luminex, TX, USA).

### Guinea pig experiments.

Fifty-six guinea pigs were anesthetized by intraperitoneal injection of ketamine (20 mg/kg) and xylazine (1 mg/kg). Anesthetized guinea pigs were infected i.n. or i.t. with 300 μL of MRV3 (150 μL per nostril or testis) (1 × 10^6^ PFU). Animals were monitored daily for clinical signs of disease. On days 1, 2, 3, 4, 5, 7, and 10 after inoculation, 2 animals from each group were euthanized and lung and testes were collected. Two independent tests were performed. Additionally, at two terminal test points (3 and 7 dpi), BAL was performed and the lungs were removed to isolate the virus from the tissue homogenates.

Processed and purified oral and nasal swabs, BALF supernatants, and urine samples from infected guinea pigs were analyzed for the viral load. Briefly, nasal and oral samples were collected by gently running a sterile swab under the animal mouth and nose. Swabs were placed immediately into 1 mL of R10 medium. All samples were centrifuged for 10 min at 800 × *g* and sterile filtered (syringe filter, 0.45 μm, cellulose acetate [CA]; Sartorius). The Vero cells (1 × 10^6^) in 3 mL MEM-H with 5% FBS per well were seeded in 6-well cell culture plates (TPP, Switzerland) and inoculated with supernatant. Prepared test samples were incubated at 35°C and 5% CO_2_ for 10 days and examined daily for signs of CPE.

The close contact transmission experiments were performed according to described protocols (40, 41). The group of 16 guinea pigs was inoculated with MRV3 (1 × 10^6^ PFU per animal) by the i.n. route. The infected animals were separated in 4 cages. One naive guinea pig was cocaged on day 1 postinfection into each group, and animals were kept together until day 7 postinfection. Oral and nasal swabs from naive guinea pigs were collected every day from days 1 to 7 during cocaging with infected animals. On days 4 and 7 p.i., animals were euthanized. Lungs and trachea were taken from all naive guinea pigs at the terminal point of the test and grown in the culture for 10 days as described above.

For the estimation of humoral and cellular immune responses in MuV-infected guinea pigs, 12 6-week-old animals were divided into 4 groups: control group (which received saline solution) and 3 groups i.n. infected with different wild-type MuV strains (1 × 10^6^ PFU). Blood samples from the lateral saphenous vein were collected on days 7, 14, 21, and 28 p.i. On day 28 p.i., necropsy was performed and lung and trachea were harvested and subjected to histopathology assays.

For evaluation of protection against MuV replication in the lungs, three groups (*n* = 5 animals per group) were formed, two experimental and one control group. The experimental groups were immunized subcutaneously (s.c.) with vaccine (L-Zagreb) or recombinant MuV (MRV2) in a volume of 500 μL (1 × 10^6^ PFU/animal). The animals in the control group were injected s.c. with 500 μL of saline solution. On day 21 after the prime immunization, guinea pigs were boosted in the same manner as described above. To check the effect of protective immunity, guinea pigs were challenged on day 14 after immunization with 1 × 10^7^ PFU of MuV MRV3 via the intranasal route. Animals were euthanized 3 days postchallenge, and BALFs were harvested for detection of viral load.

### IFN-γ ELISpot assay.

Ninety-six-well multiscreen HTS plates (Millipore, Billerica, MA) were coated with 100 μL primary anti-IFN-γ antibody solution per well (5 μg/mL in PBS, pH 7.4, V-E4) for 24 h at 4°C. The second day after washing with 250 μL PBS, specific binding was blocked with 200 μL blocking buffer (10% sucrose and 2% BSA in PBS) for 2 h at RT. After blocking and washing, 1 × 10^5^ splenocytes in 100 μL of RPMI were mixed with 50 μL stimulant (100 μL of no-stimulation dimethyl sulfoxide [DMSO] control [PBS-5% DMSO], positive control concanavalin A [ConA] at 20 μg/mL, or different MuV strains at an MOI of 2) in triplicate. Unstimulated cells served as negative controls, with ConA-stimulated cells serving as a positive control. On the 3rd day after incubation at 37°C for 18 h, cells were removed by washing and 100 μL of biotinylated secondary anti-IFN-γ antibody (2 μg/mL, N-G3) in blocking buffer was added to each well. Following 2 h of incubation and washing, alkaline phosphatase-conjugated streptavidin (SEL002; R&D Systems Inc., Minneapolis, MN) was diluted 1:100, and then 100 μL was added to each well for 1 h at RT. Following washes, cells were incubated for 20 min at RT with 100 μL of BCIP-NBT (5-bromo-4-chloro-3-indolylphosphate–nitroblue tetrazolium) detection reagent (SEL002; R&D Systems Inc., Minneapolis, MN), and spots were counted under a microscope (Zeiss Stemi 508 Greenough stereomicroscope; Zeiss, Germany). To ensure accuracy and reliability of the data, two independent operators performed the counting. Mouse monoclonal anti-guinea pig IFN-γ antibodies (N-G3 and V-E4) for guinea pig IFN-γ ELISpot assay were kindly provided by Hubert Schafer, Robert Koch Institute, Berlin, Germany.

### ELISA.

Humoral responses were monitored by ELISA as previously described (48) with some modifications. High-binding 96-well plates (Costar, USA) were coated with 100 μL of ultracentrifuged MuV (vaccine strain) in carbonate buffer (pH 9.6) and left overnight at RT. The next day, after washing and blocking with 2% BSA in PBS/T (0.05% Tween 20 in PBS) buffer (200 μL/well) for 2 h at 37°C, the investigated sera (2-fold diluted) were added in duplicates and incubated for the next 2 h. After washing, plates were incubated with anti-guinea pig IgG-horseradish peroxidase (HRP) for 2 h at 37°C. Finally, after washing, the *o*-phenylenediamine (OPD) solution (0.6 mg/mL in citrate-phosphate buffer, pH 5.0) containing 30% H_2_O_2_ (0.5 μL/mL of OPD solution) was added and incubated for 30 min at RT in the dark. The enzymatic reaction was stopped with 12.5% H_2_SO_4_ (50 μL/well), and absorbance was measured at 492 nm using a microplate reader (BioTek Synergy HTX multimode reader; Agilent, USA). The quantity of anti-mumps virus immunoglobulin G (IgG) in guinea pig sera was determined by parallel line assay comparing each serum with positive serum (declared to 100 arbitrary units [AU]/mL) and negative-control serum from nonimmunized guinea pig. The results are presented in arbitrary units per milliliter.

### Flow cytometry.

Blood cells were hemolyzed by adding RBC lysis buffer (BioLegend, USA). The cells were washed twice and resuspended in a PBS staining buffer with 1% BSA. To collect BALF, the trachea was cannulated, the lungs were lavaged with 3-mL fractions of 10 mM PBS with EDTA, and cells from BALF were centrifuged and resuspended in the staining buffer (PBS with 1% BSA). Prepared whole blood and cells from BALF were stained with the following mouse monoclonal antibodies (MAbs): anti-guinea pig CD45 (IH-1; Bio-Rad, Hercules, CA, USA), anti-guinea pig CD8 (CT6; Bio-Rad), anti-guinea pig CD4 (CT7; Bio-Rad), anti-guinea pig B cells (Msgp9; Bio-Rad), anti-guinea pig T cells (Msgp7; Bio-Rad), and anti-porcine granulocyte (MIL4; Bio-Rad). Cell suspensions from each individual guinea pig were incubated with the antibodies at 4°C for 30 min in the dark, and then cells were washed with PBS containing 1% BSA. Data acquisition and analysis were done using a Guava easyCyte flow cytometer (Luminex). Analyses were performed with an acquisition of at least 100,000 total events. Guinea pig leukocytes were initially gated into 4 fractions by flow cytometry using MIL4/side scatter (SSC) parameter (44). The lymphocyte subpopulations were further studied with the adjusted lymphocyte population using various guinea pig antibodies as described above.

### Analysis of complement system activation.

The hemolytic activity of complement (the classical pathway method, CH50 [the CH50 measures the total hemolytic activity of a test sample and is the reciprocal of the dilution of serum complement needed to lyse 50% of a standardized suspension of sheep erythrocytes coated with antierythrocyte antibody]) was used to assess the inhibition or consumption of complement components in infected guinea pigs. Guinea pig sera from control and infected animals were collected on day 3 p.i. and immediately stored at −80°C in small aliquots until analysis. A commercial kit (CH50 test; HaemoScan, Netherlands) was used according to the manufacturer’s recommendations.

### Tissue preparation and histological examination.

For histopathology, the lung samples were collected from infected guinea pigs on day 28, and testicles were collected on day 7 p.i. The samples were fixed in 4% neutral buffered formalin (Claro-Prom d.o.o., Zagreb, Croatia) embedded in paraffin, sectioned, and HE stained.

### Statistical analysis.

Statistical analyses were performed using GraphPad Prism v.9.3.1. A two- or one-way analysis of variance (ANOVA) followed by Tukey’s multiple-comparison test was used for normally distributed data. A two-tailed Mann-Whitney U test or a Kruskal-Wallis test followed by Dunn’s *post hoc* test was used when data were not normally distributed. Significance is indicated as follows: *, *P* < 0.05; **, *P* < 0.01; ***, *P* < 0.001; and ****, *P* < 0.0001.

### Ethical statements.

Animal experimentation was approved by the Croatian Ministry of Agriculture, Veterinary and Food Safety Directorate (UP/I-322-01/17-01/74, permission no. 525-10/1315-20-11, date 29 April 2020). The approval was based on the positive opinion of the National Ethical Committee (EP 109/2017). The protocols for animal care and handling were in accordance with the guidelines of the Croatian Law on Animal Welfare (2017), which strictly complies with EC Directive 2010/63/EU. All animal procedures were performed by fully trained and licensed staff, with obtained education and training according to the category B or C of the Federation of European Laboratory Animal Science Associations.
