# Supplementary material for: Evaluation of the Interactions between Mumps Virus and Guinea Pig
Source: J Virol. 2023 Apr 5;97(4):e00359-23. doi: 10.1128/jvi.00359-23 (PMC10134796; doi:10.1128/jvi.00359-23)

Supplementary methods

Evaluation of the interactions between mumps virus and guinea pig

Running Head: Mumps virus in guinea pigs

Maja Lang Balija[a,b]#, Adela Štimac[a,b], Tanja Košutić Gulija[a,b], Andrea Gudan Kurilj[c], Ana Bekavac[d], Ante Plećaš[e], Beata Halassy[a,b], Maja Jagušić[a,b], Dubravko Forčić [a,b]#

[a]Centre for Research and Knowledge Transfer in Biotechnology, University of Zagreb, Zagreb, Croatia

[b]Center of Excellence for Viral Immunology and Vaccines, CERVirVac, Zagreb, Croatia

[c]Department of Veterinary Pathology, Faculty of Veterinary Medicine University of Zagreb, Croatia

[d]Laboratory for Stem Cells, School of Medicine, Zagreb, Croatia

[e]Department of Anatomy, Histology and Embryology, Faculty of Veterinary Medicine University of Zagreb, Zagreb, Croatia

#Address correspondence to Maja Lang Balija (mlbalija@unizg.hr) and Dubravko Forčić (dforcic@unizg.hr)

Maja Lang Balija and Dubravko Forčić contributed equally to this work. Author order was determined on the basis of expertise in animal handling and performing *in vivo* experiments.

Table S1 List of primary antibodies used in the study.

antibody	clone	species	source	cat. number	dilution
GFAP		chicken IgY	Abcam	ab4674	1:250
Nestin	rat-401	mouse IgG	Merck (Sigma)	MAB353	1:200
S100B	CL2720	mouse IgG1	Merck (Sigma)	AMAb91038	1:500
Vimentin	EPR3776	rabbit IgG	Abcam	ab92547	1:250

Table S2 List of secondary antibodies used in the study.

antibody	conjugate	source	cat. number	dilution
Donkey anti-Mouse IgG (H+L)	Alexa Fluor 488	ThermoFisher Scientific	A-21202	1:1000
Donkey anti-Mouse IgG (H+L)	Alexa Fluor 555	ThermoFisher Scientific	A-31570	1:1000
Donkey anti-Rabbit IgG (H+L)	Alexa Fluor 546	ThermoFisher Scientific	A-10040	1:1000
Goat anti-Chicken IgY (H+L)	Alexa Fluor 488	ThermoFisher Scientific	A-11039	1:1000

FIG S1 Representative HPLC chromatogram of released DMB-labelled SAs after treatment of guinea pig primary lung cells with α2,3-sialidase. Peaks: Neu5Gc (t = 8.929 min); Neu5Ac (t = 10.563 min).

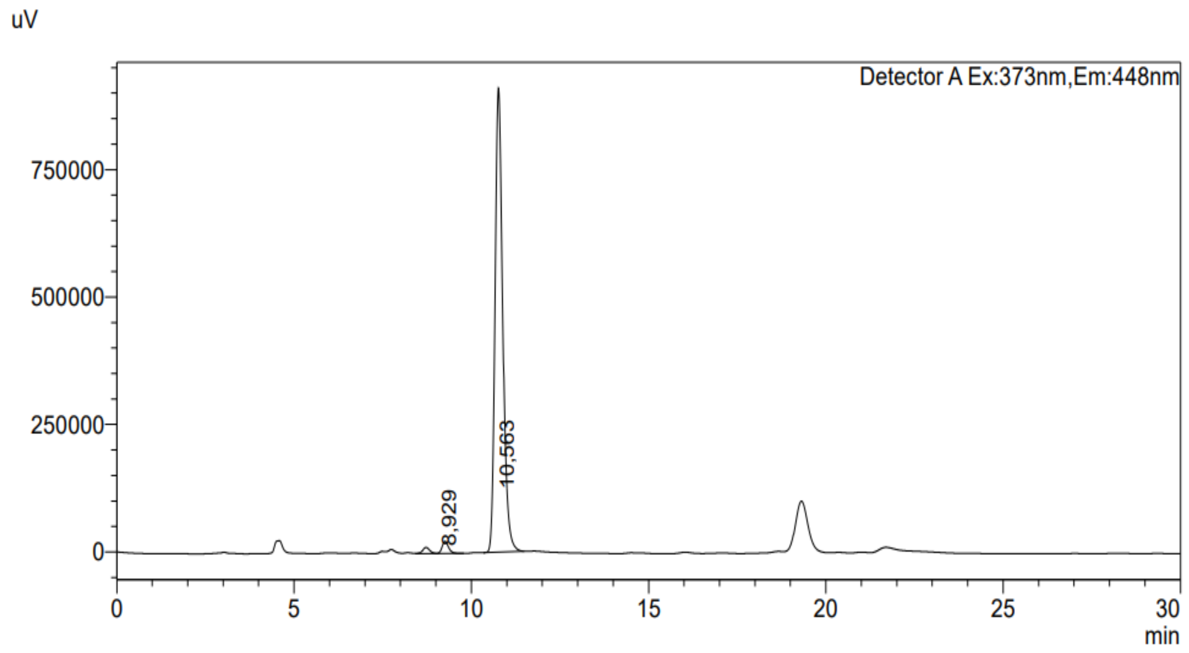

FIG S2 Released Neu5Ac during optimisation of conditions for sialidases treatment of guinea pig primary lung cells.

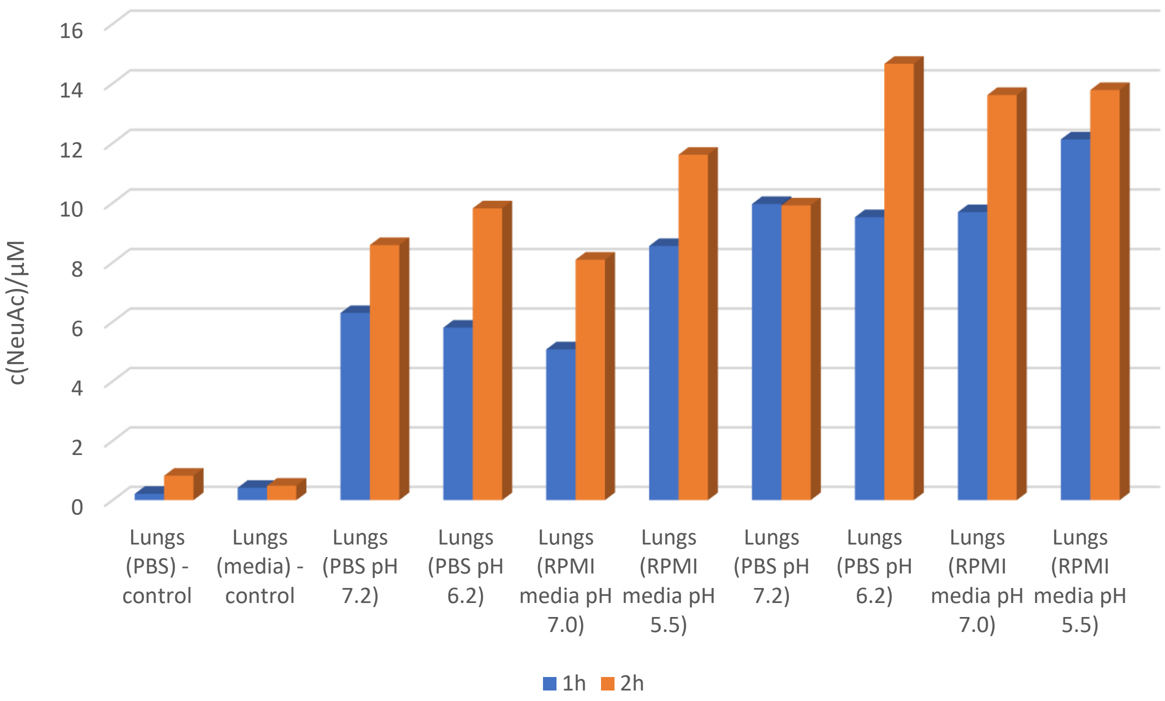

Supplement: Supplemental file 1 — Tables S1 and S2 and Fig. S1 and S2. Download jvi.00359-23-s0001.pdf, PDF file, 0.2 MB [file jvi.00359-23-s0001.pdf]
